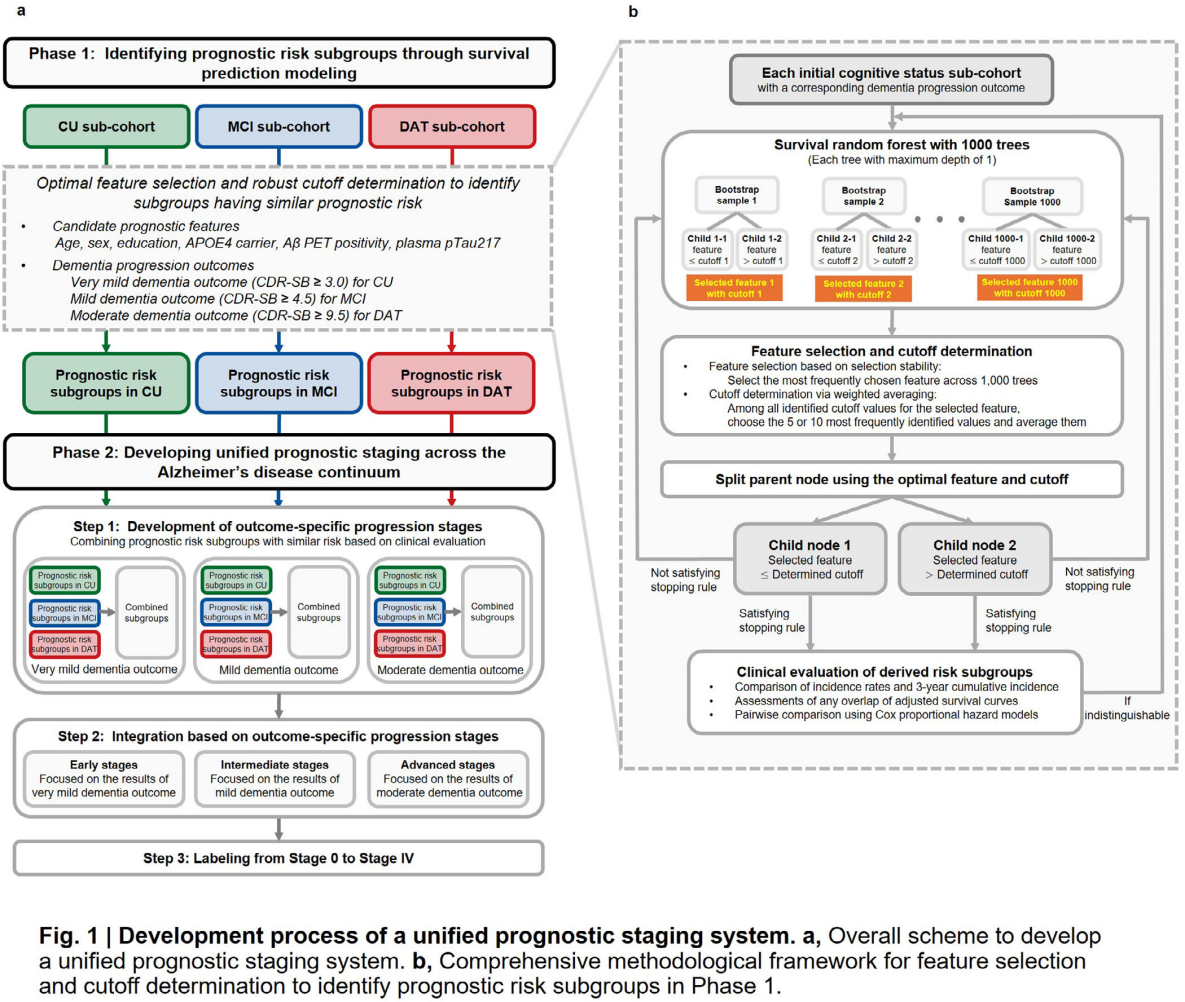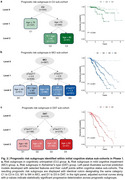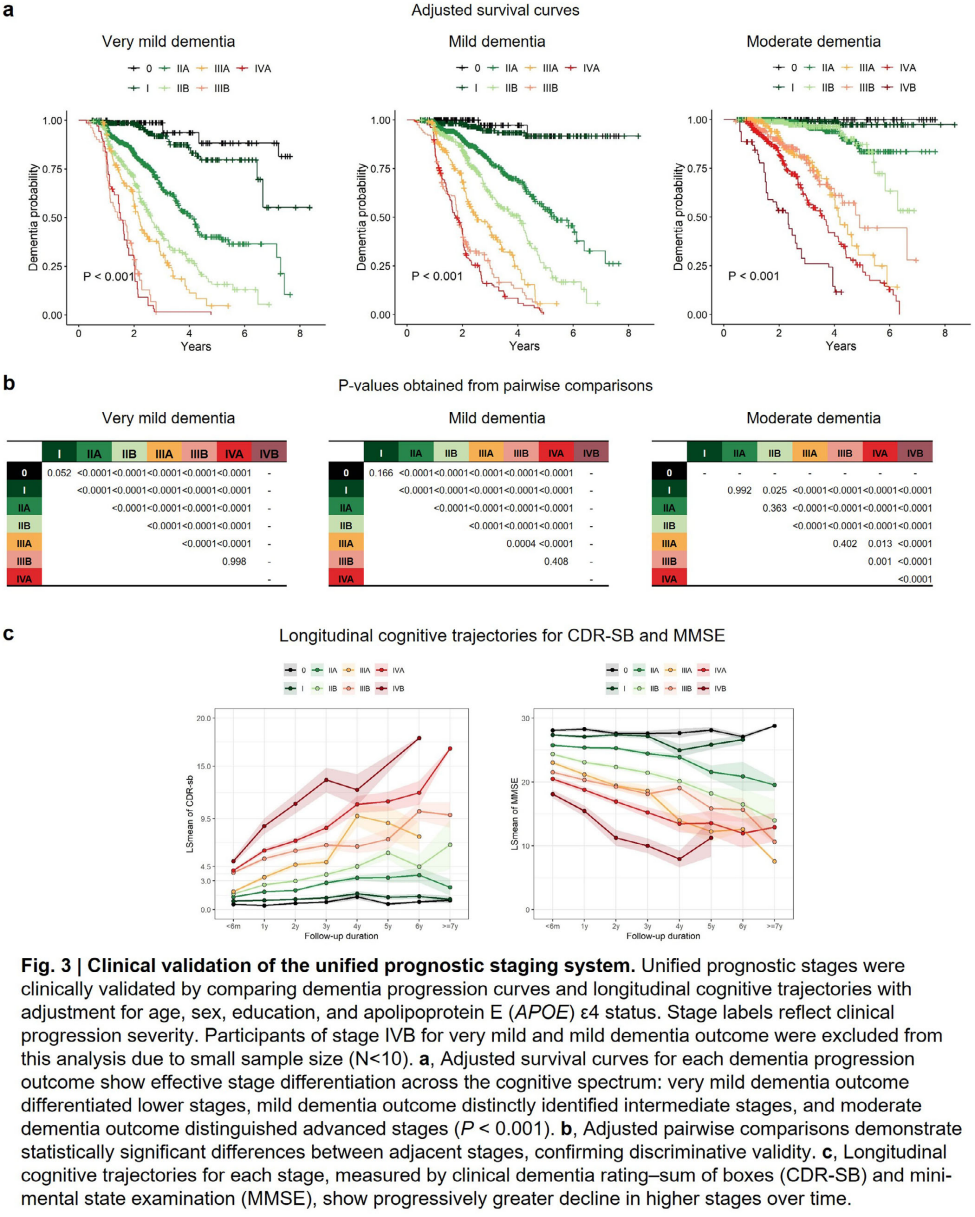# Biomarker‐integrated Prognostic Stagings for Alzheimer's Disease

**DOI:** 10.1002/alz70861_108341

**Published:** 2025-12-23

**Authors:** Daeun Shin, Sungjoo Lee, Junpyo Kim, Hyemin Jang, Jihwan Yun, Min Young Chun, Jehyun Ahn, Seongmi Kim, Kyoungmin Kim, Soyeon Yoon, Hee Jin Kim, Heekyoung Kang, Sohyun YIM, Hee Kyung Park, Sunghyun Kim, Duk L Na, Henrik Zetterberg, Kaj Blennow, Fernando Gonzalez‐Ortiz, Nicholas Ashton, Michael S. W. Weiner, Sang Won Seo, Kyunga Kim, ADNI, K‐ROAD

**Affiliations:** ^1^ Samsung Medical Center, Gangnam‐gu, Seoul Korea, Republic of (South); ^2^ Research Institute for Future Medicine, Samsung Medical Center, Gangnam‐gu, Seoul Korea, Republic of (South); ^3^ Seoul National University Hospital, Seoul National University College of Medicine, Jongno‐gu, Seoul Korea, Republic of (South); ^4^ Kyung Hee University Medical Center, Seoul Korea, Republic of (South); ^5^ Yongin Severance Hospital, Yonsei University Health System, Yongin, Gyeonggi‐do Korea, Republic of (South); ^6^ Samsung Medical Center, Gangnam‐Gu, Seoul Korea, Republic of (South); ^7^ Samsung Medical Center, Seoul, Ilwon‐ro Korea, Republic of (South); ^8^ Samsung Medical Center, Sungkyunkwan University School of Medicine, Seoul, Ilwon‐ro Korea, Republic of (South); ^9^ Clinical Neurochemistry Laboratory, Sahlgrenska University Hospital, Mölndal, Västra Götalands län Sweden; ^10^ Clinical Neurochemistry Laboratory, Sahlgrenska University Hospital, Mölndal, Gothenburg Sweden; ^11^ Department of Psychiatry and Neurochemistry, Institute of Neuroscience and Physiology, The Sahlgrenska Academy, University of Gothenburg, Mölndal Sweden; ^12^ Memory and Aging Center, Weill Institute for Neurosciences, University of California San Francisco, San Francisco, CA USA; ^13^ Samsung Medical Center, Sungkyunkwan University School of Medicine, Seoul Korea, Republic of (South)

## Abstract

**Background:**

Accurate staging of Alzheimer’s disease (AD) progression is crucial for optimizing patient care and therapeutic interventions; however, traditional cognition‐based classifications remain limited to capture the heterogeneity in individual risk.

**Methods:**

A total of 1,738 participants from a Korean multi‐center cohort, including cognitively unimpaired (CU), mild cognitive impairment (MCI), and dementia of Alzheimer’s type (DAT), were first stratified into prognostic risk subgroups according to cognitive status using random survival forest models (Figure 1). Candidate prognostic predictors included age, sex, education years, APOE ε4 status, plasma phosphorylated tau 217 (pTau217) and amyloid PET positivity. Dementia progression was defined by clinical dementia rating‐sum of boxes (CDR‐SB) thresholds. These prognostic subgroups were unified into eight prognostic stages, ranging from Stage 0 to Stage IVB based on pairwise comparisons and incidence metrics. External validation was performed using 370 participants from the Alzheimer’s Disease Neuroimaging Initiative cohort.

**Results:**

Prognostic predictors varied by cognitive status, with plasma pTau217 consistently outperforming amyloid PET (Figure 2). Age increased dementia risk in CU and MCI, while younger age was associated with faster progression in DAT. The biomarker‐integrated staging system reclassified individuals beyond cognitive labels, with distinct prognostic trajectories (log‐rank test for adjusted survival curves, all *p* <0.0001; pairwise comparisons, majority *p* <0.0001) (Figure 3). Higher stages were significantly associated with increased dementia risk, faster CDR‐SB progression, and lower mini‐mental state examination scores. External validation confirmed the discriminative power and generalizability of the staging system.

**Conclusion:**

A unified staging system integrating cognitive status, plasma pTau217, and age effectively stratifies AD progression risk and predicts cognitive decline. This framework provides a clinically actionable model to guide personalized treatment strategies and monitor therapeutic efficacy, especially in the era of disease‐modifying therapies.